# Effects of Herbst and Pendex appliances treatment on the upper airway: A cone-beam computed tomography analysis

**DOI:** 10.1016/j.ajodo.2026.01.019

**Published:** 2026-03-16

**Authors:** Ward Kyle, Jiahui Madelaine Li, Brianna Tucker, Sam Kadan, Andrew S. Wiemken, Bruno Saconi, Brendan T. Keenan, Richard J. Schwab, Chun-Hsi Chung, Hyeran Helen Jeon

**Affiliations:** a School of Dental Medicine, University of Pennsylvania, Philadelphia, Pa; b Department of Orthodontics, School of Dental Medicine, University of Pennsylvania, Philadelphia, Pa; c Private Dental Orthodontic Practice, Chalfont, Pa; d Division of Sleep Medicine, Department of Medicine, University of Pennsylvania Perelman School of Medicine, Philadelphia, Pa

## Abstract

**Introduction:**

Class II functional appliances are widely used to treat skeletal Class II malocclusion in growing patients, whereas molar distalization is often employed for dental Class II malocclusion. However, the impact of these treatment modalities on upper airway dimensions remains controversial. This study examines the effects of Herbst appliance treatments on the upper airway changes compared with the Pendex appliance in adolescent patients with Class II malocclusion.

**Methods:**

Cone-beam computed tomography (CBCT) scans of 50 patients with Class II malocclusion were obtained from a private orthodontic office (Chalfont, Pa). Twenty-five patients were treated with Herbst appliances (12.16 ± 1.89 years; 13 males and 12 females) and 25 patients with Pendex appliances (11.72 ± 1.22 years; 12 males and 13 females). Measurements were taken for volume, length, and cross-sectional areas of the total, retropalatal, and retroglossal upper airway. CBCTs were taken at baseline (T1), after appliance removal (T2), and from final records after fixed edgewise appliance treatment (T3) (T1-T3: 41.11 ± 9.95 months for Herbst appliances and 44.81 ± 9.63 months for Pendex appliances). In addition to 3-dimensional CBCT analysis, 2-dimensional measurements were performed and compared with a Class II untreated control group comprising 25 age- and sex-matched patients from the American Association of Orthodontists Foundation Bolton-Brush Craniofacial Growth Legacy Collection. Differences across time points within groups were assessed using repeated-measures analysis of variance test, whereas between-group differences at individual time points and for changes from T1 to T3 were evaluated using independent-samples *t* tests adjusted for baseline (T1) outcome values and follow-up duration.

**Results:**

From T1 to T3, both the Herbst and Pendex groups showed a significant increase in total upper airway volume and length. However, the magnitude of these changes did not differ significantly between the 2 groups. In the 2-dimensional analysis, no significant differences were observed among the Herbst, Pendex, and untreated control groups in airway area changes from T1 to T3.

**Conclusions:**

Both the Herbst and Pendex appliances resulted in comparable increases in total upper airway volume and length in adolescent patients with Class II malocclusion, primarily attributable to normal growth.

Skeletal Class II malocclusion with a retrognathic mandible is a common orthodontic condition, affecting approximately 20% of the global population with permanent dentition.^1^ Both removable and fixed functional appliances, such as the Twin-block, Herbst appliance, and mandibular anterior repositioning appliance (MARA), are widely used to treat adolescent patients with skeletal Class II malocclusion. These appliances facilitate anterior mandibular positioning and encourage adaptive changes in the bone and muscles.^2^ Among them, the Herbst appliance is currently the most commonly used device for mandibular advancement in growing patients with Class II malocclusion in the United States.^3^ However, the extent to which functional appliances induce condylar remodeling and promote mandibular growth remains a topic of debate.^4–9^

Skeletal discrepancies between the maxilla and mandible in the sagittal dimension, particularly mandibular retrognathism, are linked to oropharyngeal airway constriction, and they may contribute to the development of obstructive sleep apnea syndrome.^10,11^ Forward positioning of the mandible with functional appliances is associated with anterior tongue displacement and an improvement in the upper airway dimensions.^8,12–14^ Interestingly, oral devices used to treat obstructive sleep apnea share a similar design with the Herbst appliance, advancing the mandible to increase upper airway caliber.^15^ Previous studies evaluating removable functional appliances, such as activators, Twin-blocks, or bionators, have yielded controversial outcomes. Although some studies demonstrate significant improvements in upper airway dimensions in groups treated with removable functional appliances compared with controls,^16–19^ other studies report minimal or no significant changes.^20^ This variability may partially result from reliance on patient compliance, as removable appliances depend heavily on consistent use. In contrast, fixed functional appliances, such as the Herbst appliances and MARA, operate continuously and do not require patient cooperation.^21^ Studies investigating these fixed functional appliances have reported significant increases in oropharyngeal airway dimensions compared with groups without Class II malocclusion correction.^22,23^ However, these studies have primarily assessed immediate or short-term effects. In addition, some research has reported negligible changes, underscoring the variability and inconsistent outcomes associated with Class II growth modification treatments on upper airway dimensions.^24,25^ At present, robust evidence supporting the long-term positive effects of Herbst appliance therapy on the upper airway dimensions in adolescent patients with Class II malocclusion remains limited.

Maxillary molar distalization, using the Pendulum, Pendex, distal jet, and modified Nance appliance, is considered an effective treatment option for patients with mild Class II malocclusion.^26^ Although treatment with functional appliances can achieve both skeletal and dental improvements in the maxillomandibular relationship, Class II malocclusion correction through maxillary molar distalization primarily results in dentoalveolar changes. Generally, molar distalization is associated with an increase in the mandibular plane angle, leading to mandibular clockwise rotation.^27^ Previous studies suggest that the vertical changes induced by molar distalization have a negligible impact on the upper airway and that this treatment is not associated with long-term airway dimensional changes in adolescent patients.^28,29^

This study aims to evaluate the upper airway changes in adolescent patients with Class II malocclusion treated with the Herbst appliance vs those treated with molar distalization using the Pendex appliance. We hypothesize that treatment with the Herbst appliance will lead to a greater increase in oropharyngeal airway dimensions compared with the Pendex appliance.

## MATERIAL AND METHODS

The retrospective study was approved by the institutional review board at the University of Pennsylvania (Protocol No. 851012). This study analyzed cone-beam computed tomography (CBCT) images from 50 patients who consecutively received either Herbst or Pendex appliance treatment between October 2016 and January 2023 at a private orthodontic office in Chalfont, Pennsylvania. Patients with a history of previous orthodontic treatment, permanent tooth extractions, or craniofacial surgeries before undergoing Herbst or Pendex appliance treatment were excluded. The study sample was divided into 2 groups (Table I).

Herbst group (growth modification group): 25 patients (aged 12.2 ± 1.9 years at T1; 13 males and 12 females) treated with Herbst appliances, followed by fixed orthodontic appliances. CBCT scans were acquired in the natural head position at preorthodontic treatment records (T1), 6 months after Herbst appliance removal (T2, aged 14.6 ± 2.1 years), and posttreatment final records after the removal of fixed edgewise appliances (T3, aged 15.6 ± 1.9 years). The time period from T1 to T2 was 29.2 ± 9.8 months, from T2 to T3 was 11.9 ± 7.9 months, and from T1 to T3 was 41.1 ± 10.0 months.

Pendex group (nongrowth modification control): 25 patients (aged 11.7 ± 1.2 years at T1; 12 males 13 females) treated with Pendex appliances, followed by fixed orthodontic appliances. CBCT scans were acquired in the natural head position at preorthodontic treatment records (T1), immediately after the removal of Pendex (T2, aged 13.0 ± 1.6 years), and posttreatment final records after the removal of fixed edgewise appliances (T3, aged 15.5 ± 1.2 years). The time period from T1 to T2 was 14.8 ± 8.2 months, from T2 to T3 was 30.0 ± 13.4 months, and from T1 to T3 was 44.8 ± 9.6 months.

Our sample size of 25 patients per group is relatively modest, with 80% power to detect a large effect size (0.80) in 2-group analyses and a moderate effect size (0.58) in paired analyses at an α = 0.05.

After the initial orthodontic records, patients in the Herbst group received a stainless-steel crown Herbst appliance, along with an expander and a lower lingual holding arch. For all patients in this study, patients and parents were instructed to carry out 1 turn per day (0.25 mm per turn) for 30-35 days. After expansion, the telescopic arms were placed, and the Herbst appliance was activated until the patient achieved an anterior edge-to-edge bite, which was maintained for at least 1 year. For the Pendex group, the Pendex appliance was delivered with bands on the maxillary first molars and occlusal rests on the premolars or primary molars. The maxillary expansion was completed with 30-35 turns. After the distalization of the maxillary first molars was completed, a Nance holding arch was placed for retention. For both groups, the fixed edgewise appliance treatment were followed. Based on the treatment protocols, the T1-T2 periods differed between the 2 groups. However, the T1-T3 time periods were similar, so we focused on the differences at T1, T3, and the T3-T1 changes.

**Fig 1.**
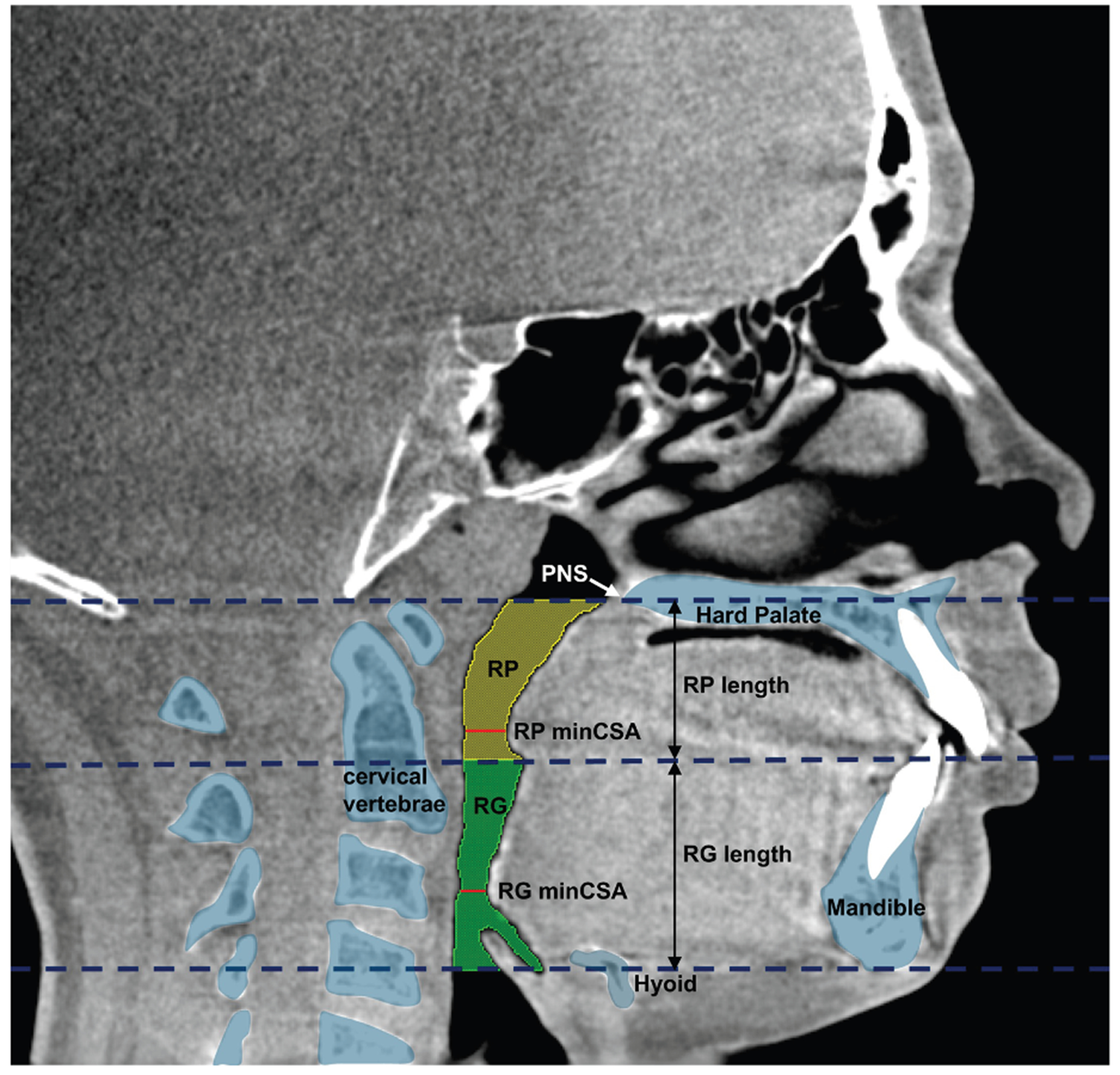
The upper airway measurements. *PNS*, posterior nasal spine.

CBCT scans were obtained in natural head position.^30–32^ Patients were instructed to keep their mouths closed, breathe through the nose, bring the teeth into light contact, and swallow once. From the T1 and T3 CBCTs, lateral cephalograms were generated as an orthogonal view (0% magnification) of the right side of the face to the midpoint of the maxillary left central incisor, as previously described.^33^ Digital tracing and cephalometric measurements of ANB and SN-GoGn were performed using the Dolphin Imaging & Management Solutions software (version 12.0; Dolphin Imaging & Management Solutions, Chatsworth, Calif). Then, the Digital Imaging and Comunications in Medicine files were imported into the Amira software (version 5.3.0; Thermo Fisher Scientific, Waltham, Mass). CBCT images were oriented in the frontal and lateral views. In the frontal view, the inferior orbital rims were positioned symmetrically and parallel to the floor, and the midsagittal plane was defined by the nasion, anterior nasal spine, and midpoint of the chin. In the lateral view, the Frankfort horizontal plane was oriented parallel to the floor. Additional orientation adjustments were performed to optimize bilateral alignment of the orbital rims and the posterior borders of the mandibular rami. Brightness and contrast were standardized by setting the display range to −750 to 500 in the Zoom and Data window. Each scan was segmented into the retropalatal (RP) and retroglossal (RG) regions using boundary definitions on the midsagittal slice, as previously described (Fig 1).^34–37^ The boundaries of each region are outlined in Table II.^38^ In the axial view, each 0.3 mm slice was individually segmented to remove artifacts and ensure accurate tracing of airway boundaries. After segmentation, volume, length, and mean and minimum cross-sectional areas (CSA) were calculated for the total upper airway and in the RP and RG regions.

Twenty-five age- and sex-matched untreated patients with Class II malocclusion (aged 12.0 ± 0.7 years at T1; 13 males and 12 females) were selected from the American Association of Orthodontists Foundation Bolton-Brush Craniofacial Growth Legacy Collection. Two-dimensional (2D) lateral cephalometric radiographs were obtained at T1 and T3 timepoints, with image magnification standardized using fiducial landmarks. At T1, the control group had an ANB of 5.0° ± 1.96° and an SN-GoGn of 29.61° ± 4.46°, with no significant differences compared with the corresponding skeletal values in the Herbst group. The average duration between T1 and T3 was 41.4 ± 8.2 months. After orientation of the lateral cephalometric radiographs, the area, length, and mean and minimum anterioposterior (AP) distances were measured for the total upper airway, as well as for the RP and RG regions, using boundaries consistent with those applied in the corresponding 3-dimensional (3D) measurements (Fig 1).

## STATISTICAL ANALYSIS

All measurements were carried out by 1 examiner (W.K.) in a blinded fashion. To verify the consistency and reliability of the measuring protocol, the CBCT images of 10 patients were randomly selected and remeasured by the same examiner at least 1 month after initial measurements. The intraclass correlation coefficient (ICC) of the measurements was calculated using SPSS software (version 26.0; IBM, Chicago, Ill). Using guidelines provided by Landis and Koch,^39^ ICC values can be interpreted as poor (ICC <0.00), slight (0.00-0.20), fair (0.21-0.40), moderate (0.41-0.60), substantial (0.61-0.80), and almost perfect (0.81-1.00) reliability.

Participant characteristics were summarized as means and standard deviations for continuous traits or frequencies and percentages for categorical traits. Mean percentage change from the baseline (T1) to postintervention time points (T2 and T3) was calculated to describe within-group change magnitudes. Baseline between-group differences in continuous variables were assessed using independent-samples *t* tests or Mann-Whitney U tests (3D metrics; Herbst vs Pendex only) and Welch 1-way analysis of variance (ANOVA) (2D metrics; Herbst, Pendex, and untreated control). Categorical variables (eg, sex) were compared using chi-square tests. Parametric assumptions were examined by visual inspection of histograms and the Shapiro-Wilk test for normality and by tests of homogeneity of variance (eg, Levene-type tests).

To evaluate longitudinal change in 3D measures within the Herbst and Pendex groups, repeated-measures ANOVA or the Friedman test (repeated-measures ANOVA on ranks) was applied. When omnibus tests were significant, pairwise comparisons between time points were conducted. For repeated-measures ANOVA models, differences between estimated marginal means at each time-point pair were obtained from linear mixed-effects modeling. For nonparametric analysis, Friedman tests were followed by Wilcoxon signed-rank tests. Additional linear regression models (analysis of covariance) were fitted to evaluate differences in 2D variables among the Herbst, Pendex, and untreated control groups at T3, as well as changes from T1 to T3. These models were adjusted for the baseline (T1) outcome values and the duration of follow-up (ie, the interval between T1 and T3 in months) to account for initial differences and variations in treatment duration. For 2D metrics, pairwise differences in adjusted means (Herbst vs Pendex, Herbst vs control, and Pendex vs control) were derived from model contrasts after a significant omnibus group test.

To control the family-wise error rate at 5% for multiple between-group comparisons of airway measurements, the Hochberg step-up procedure was applied. Statistical significance was determined based on the Hochberg-adjusted α thresholds.^40,41^ Effect sizes were reported to describe the magnitude of group differences, using Cohen *d* for parametric contrasts (small: |0.2|, medium: |0.5|, and large: |0.8|) or Cliff delta (δ) for nonparametric contrasts (small: |0.15|, medium: |0.33|, and large: |0.47|).^42^ All statistical analyses were conducted using Stata/SE (version 16.1; Stata-Corp, College Station, Tex).

## RESULTS

The ICC of the 10 samples ranged 0.99-1.00, indicating almost perfect reliability of the measuring protocol when applied to the same image >1 month apart.

The sample in this study showed no statistically significant differences in sex, age, or vertical skeletal pattern (SN-GoGn) at T1 (Table I). However, ANB differed significantly between the 2 groups at T1, with the Herbst group exhibiting a mean ANB angle of 5.20° ± 1.89° compared with 3.47° ± 1.83° in the Pendex group (*P* = 0.002). At T3, both the vertical (SN-GoGn) and sagittal skeletal patterns (ANB) were comparable between the 2 groups. The total treatment duration from T1 to T3 showed no statistically significant difference between the Herbst and Pendex appliance groups.

**Fig 2.**
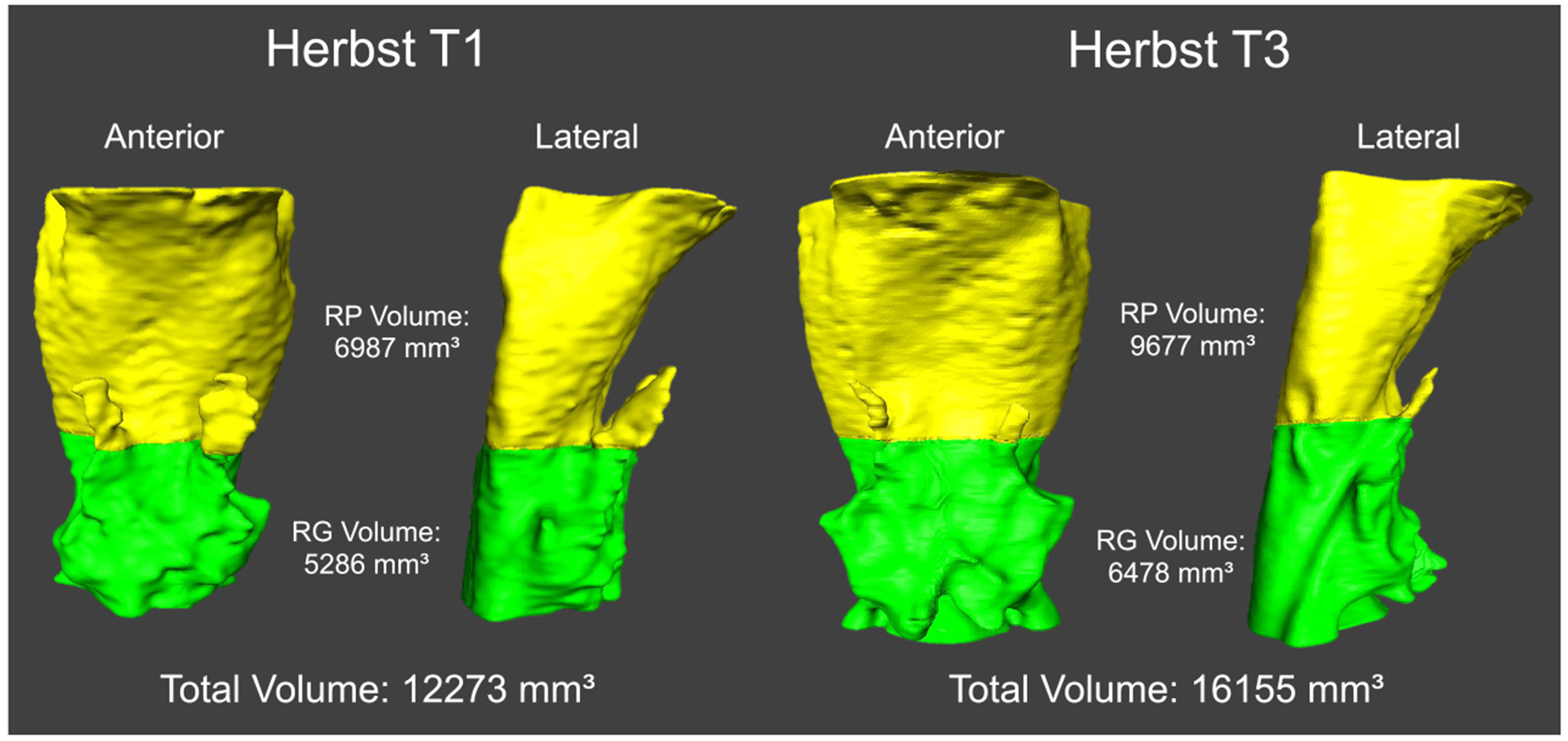
The average upper airway dimensional changes before and after Herbst appliance treatment.

In the Herbst group, from T1 to T2, significant increases were observed in total volume (27.7%, 12,273 ± 4625 mm^3^ to 15,668 ± 4659 mm^3^), total length (13.1%, 51.98 ± 5.19 mm to 58.79 ± 5.54 mm), RP volume (32.7%, 6987 ± 2772 mm^3^ to 9272 ± 3530 mm^3^), RP length (14.7%, 26.81 ± 4.21 mm to 30.76 ± 4.76 mm), RP mean CSA (15.8%, 258.20 ± 91.45 mm^2^ to 299.00 ± 93.15 mm^2^), and RG length (11.3%, 25.18 ± 6.02 mm to 28.03 ± 4.85 mm) (Table III). From T2 to T3, values were maintained or slightly increased across all measurements, with no statistically significant changes observed in the upper airway dimensions. In addition, these changes from T1 to T2 were perpetuated to T3 and led to the same pattern of differences. From T1 to T3 in the Herbst group, significant increases were noted in total volume (31.6%, 12,273 ± 4625 mm^3^ to 16,155 ± 5979 mm^3^), total length (15.5%, 51.98 ± 5.19 mm to 60.01 ± 6.33 mm), RP volume (38.5%, 6987 ± 2772 mm^3^ to 9677 ± 4480 mm^3^), RP length (16.2%, 26.81 ± 4.21 mm to 31.15 ± 5.65 mm), RP mean CSA (18.6%, 258.20 ± 91.45 mm^2^ to 306.20 ± 120.80 mm^2^), and RG length (14.6%, 25.18 ± 6.02 mm to 28.86 ± 5.93 mm) (Fig 2).

**Fig 3.**
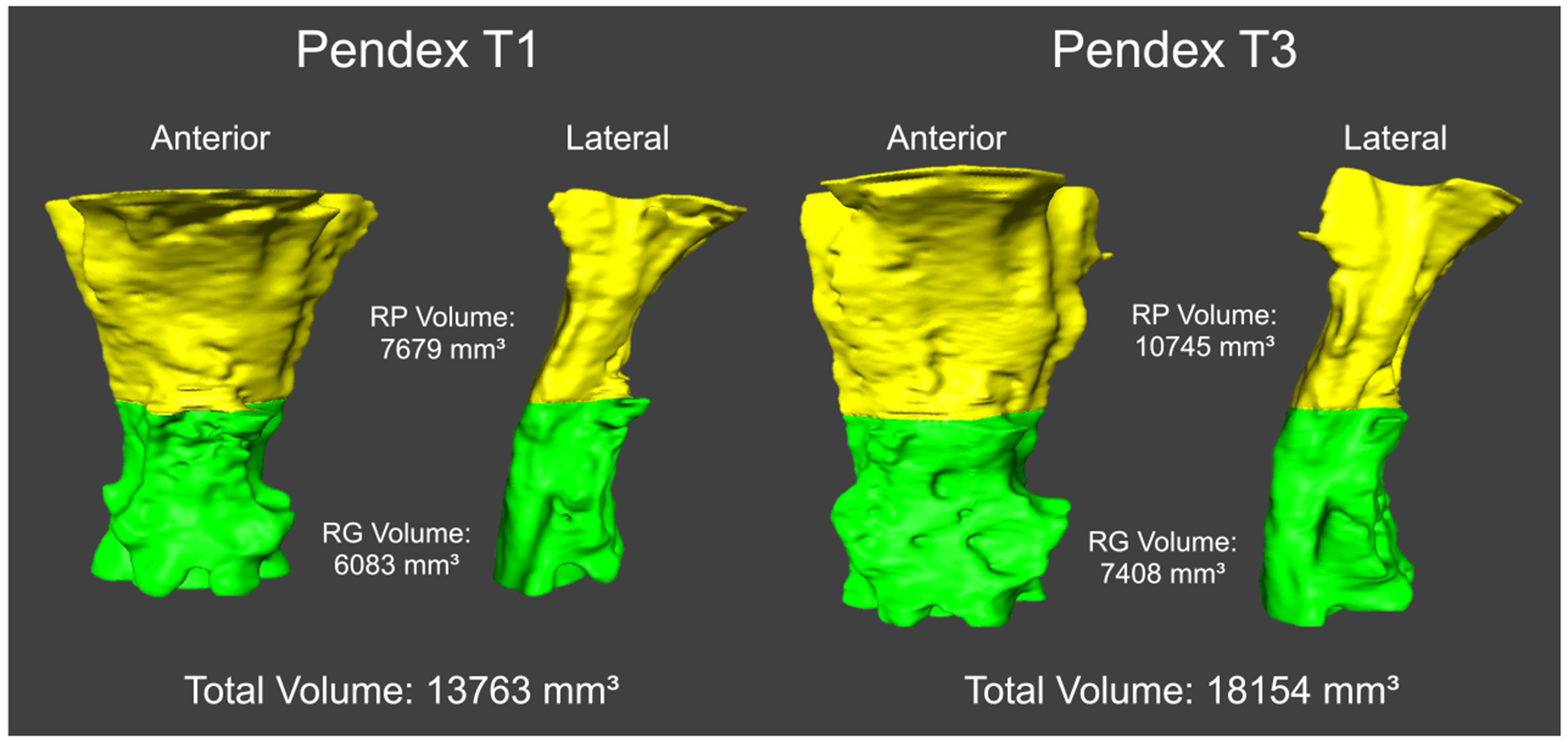
The average upper airway dimensional changes before and after Pendex appliance treatment.

In the Pendex group, no significant changes were observed in any airway dimensions from T1 to T2 (Table IV). From T2 to T3 in the Pendex group, significant increases were observed in total volume (31.3%, 13,831 ± 6086 mm^3^ to 18,154 ± 7422 mm^3^), total length (6.2%, 55.07 ± 5.18 mm to 58.48 ± 5.75 mm), total mean CSA (43.2%, 251.60 ± 110.00 mm^2^ to 307.80 ± 110.40 mm^2^), RP volume (35.9%, 7908 ± 3167 mm^3^ to 10745 ± 4499 mm^3^), RP mean CSA (24.8%, 284.20 ± 111.00 mm^2^ to 354.60 ± 119.90 mm^2^), and RP minimal CSA (34.12%, 191.40 ± 112.80 mm^2^ to 256.70 ± 103.30 mm^2^) (Table IV). From T1 to T3 in the Pendex group, significant increases were observed in total volume (31.9%, 13,763 ± 7335 mm^3^ to 18,154 ± 7422 mm^3^), total length (7.5%, 54.40 ± 5.43 mm to 58.48 ± 5.75 mm), total mean CSA (23.29%, 249.70 ± 116.60 mm to 307.80 ± 110.40 mm), RP volume (39.9%, 7679 ± 3402 mm^3^ to 10745 ± 4499 mm^3^), RP length (8.79%, 27.86 ± 3.60 mm to 30.31 ± 5.30 mm), and RP mean CSA (27.1%, 279.00 ± 120.60 mm^2^ to 354.60 ± 119.90 mm^2^) (Table IV and Fig 3).

At T1, no statistically significant differences were observed between the Herbst and Pendex groups (Table V), although a moderate effect size was noted for total airway length (Cohen *d* = −0.45). At T3, after adjusting for the baseline (T1) values and differences in follow-up duration, most measurements similarly showed no significant differences between the groups (Table VI), but moderate effect sizes were evident in some dimensions (Cohen *d* near |0.5|), including a nominally larger RP minimal CSA in the Pendex group (adjusted mean: 253.62 mm^2^; 95% confidence interval [CI]: 216.16, 291.08) compared with the Herbst group (adjusted mean: 199.41 mm^2^; 95% CI: 161.94, 236.87; *P* = 0.048). This difference, however, did not remain statistically significant after the Hochberg adjustment. When analyzing changes from T1 to T3, after adjusting for the baseline (T1) values and differences in follow-up duration, no statistically significant differences in the upper airway measurements were identified between the groups (Table VII), although a moderate effect size was observed for total minimal CSA (Cohen *d* = −0.56; *P* = 0.061).

At T1, the control group exhibited significantly greater airway length in the total and RG regions than both the Herbst and Pendex groups. However, there were no significant differences among the Herbst, Pendex, and untreated Class II control groups in total airway area or in the RP and RG airway areas (Table VIII). At T3, after adjustment for the baseline (T1) values of the modeled variable and follow-up duration between T1 and T3, the airway length of the control group remained significantly greater in the total airway compared with the Pendex group. Conversely, the mean and minimum AP distances of the control group were significantly shorter in the total airway and RG region compared with the Pendex group. No significant differences were found in the area of total, RP, and RG airways among the 3 groups (Table IX). When evaluating changes from T1 to T3, after adjusting for the baseline values and variations in follow-up duration, the control group demonstrated a greater increase in total airway length than the Pendex group, whereas the Pendex group exhibited larger increases in total mean and minimum AP distances compared with the control group. No statistically significant differences in the area were identified among the groups for the total, RP, and RG regions (Table X).

## DISCUSSION

In our study, although we observed an increase in the airway dimensions after Herbst appliance treatment, we found that both the Herbst and Pendex appliance groups showed comparable increases in the upper airway dimensions. Consistent with our findings, Gu et al^24^ reported similar changes in the upper airway from the Herbst appliance compared with the headgear activator when assessed using lateral cephalograms. Rongo et al^25^ reported an increase in nasopharyngeal and oropharyngeal dimensions after functional appliance treatment. However, the changes were not statistically significant compared with untreated Class II controls. Our findings differ from previous studies that reported airway enhancement effects of Class II functional appliances compared with untreated patients.^14,16,21^ Rizk et al^23^ reported a significant increase in the oropharyngeal airway volume in MARA-treated patients with Class II malocclusion compared with untreated patients with Class II malocclusion at a 33.6 month interval, which is shorter than our follow-up periods (41.1 ± 10.0 months in Herbst group and 44.8 ± 9.6 months in Pendex). Similarly, Oliveira et al^22^ reported a positive change in the oropharyngeal airway dimensions after Herbst appliance treatment compared with the untreated Class II control group, but their study was limited to a 1-year observation period. Isidor et al^16^ and Iwasaki et al^21^ reported improvement in the oropharyngeal volume from functional appliances in patients with Class II malocclusion compared with untreated patients with Class I malocclusion using CBCT.^23^ Abdalla et al^14^ reported a greater increase in the upper airway volume and minimum CSA in patients with Class II malocclusion treated with Herbst appliances compared with patients with Class I malocclusion treated with edgewise appliances. Possible reasons for the varying outcomes include differences in follow-up periods, analytical methods (lateral cephalograms vs CBCT), and the characteristics of the control groups used. The mandibular growth changes might be different in patients with Class I and Class II skeletal patterns,^43^ and patients with Class II malocclusion may not have the same compensatory growth potentials and dentoalveolar correction as patients with Class II malocclusion.^21,44,45^ Thus, the patient populations who present Class II malocclusion, but did not undergo maxillomandibular growth modification would be an ideal control group to examine the treatment outcomes of Herbst appliances. Given that the sagittal position of the mandible plays a crucial role in upper airway dynamics,^46–49^ the airway changes observed in untreated patients with Class II malocclusion are likely attributable to natural growth. However, withholding orthodontic treatment in adolescents with skeletal Class II malocclusion purely for observational purposes would raise significant ethical issues. To address these limitations, while controlling for critical variables such as sex, age, and vertical growth patterns, we chose patients with Class II malocclusion treated with molar distalization as our comparative control. This group was carefully selected as they underwent treatment without intentional changes to the maxillomandibular sagittal relationship, serving as a practical pseudo-control. In addition, we incorporated the untreated Class II control group from the American Association of Orthodontists Foundation Bolton-Brush Craniofacial Growth Legacy Collection. A 2D airway analysis from T1 to T3 revealed no significant differences in the airway area of total, RP and RG regions, among the Herbst, Pendex, and untreated Class II control groups. Although 2D imaging can be a useful screening tool, particularly for evaluating the sagittal dimension of the narrowest airway, and sagittal area measurements may serve as surrogates for volumetric assessment when 3D CBCT is unavailable, a comprehensive evaluation of airway morphology is more accurately achieved with 3D CBCT-based analysis.^50,51^ Previous studies have shown that measurements obtained from orthogonal and perspective lateral cephalometric radiographs generated from 3D CBCT data are comparable to those obtained from conventional 2D radiographs.^52^

In current literature, very few studies have compared the upper airway changes resulting from Class II functional appliances with those of untreated Class II control groups, and most of these studies relied on 2D cephalometric radiographs. Pavoni et al^53^ compared the 2D cephalometric images of patients with Class II malocclusion treated with bionators or activators to the historical cephalometric images of untreated Class II controls from the American Association of Orthodontics Foundation Craniofacial Growth Legacy Collection. They reported significant favorable changes in pharyngeal dimensions after treatment with functional appliances. Cortese et al^20^ reported that patients with Class II malocclusion treated with an activator did not demonstrate significantly different airway dimensions compared with untreated Class II controls. Similarly, Rongo et al^25^ found that functional appliance treatment resulted in statistically similar changes in the nasopharyngeal and oropharyngeal dimensions compared with untreated adolescent patients with Class II malocclusion. Both Cortese et al^20^ and Rongo et al,^25^ relying on 2D cephalometric images, reported findings consistent with ours. Alhammadi et al^1^ examined CBCT images of patients treated with Twin-blocks or the Forsus fatigue resistance device, along with untreated growing female patients with Class II malocclusion.^19^ They reported that the most significant changes were observed in the oropharyngeal airway within the Twin-block group during short-term evaluation compared with both the Forsus and control groups. The untreated control group showed an increase in the oropharyngeal volume (15,289.11 ± 3562.38 mm^3^ to 15,649.29 ± 2946.67 mm^3^) (*P* = 0.231) and minimal axial area (201 ± 78.13 mm^2^ to 222.96 ± 83.53 mm^2^) (*P* = 0.005) during an observation period (unspecified). To date, no publicly available CBCT datasets exist for untreated patients with Class II malocclusion. Consequently, previous studies have relied on 2D cephalometric analyses of patients with Class I or mild Class II malocclusions treated with conventional edgewise appliances, along with historical data from untreated patients.^14,17,18,21,22,54^

At T2, 6 months after the Herbst appliance removal, we observed significant increases in the upper airway dimensions, including total volume (27.7%), total length (13.1%), RP volume (32.7%), RP length (14.7%), and RG length (11.3%) compared with pretreatment (T1 to T2, 29.2 ± 9.8 months). At T3, after completion of orthodontic treatment, the Herbst group demonstrated a significant increase of 31.6% in total volume, 15.5% in total length, 38.5% in RP volume, 16.2% in RP length, and 14.7% in RG length from T1 to T3 (41.1 ± 10.0 months). These findings corroborate the current literature on the longitudinal enhancement of the upper airway volume after functional appliance treatment based on CBCT evaluation.^16,21,23^ Previous studies have reported increases in the oropharyngeal volume ranging 27.1%-29.7% after functional appliance treatment compared with pretreatment values,^16,22^ which aligns closely with our findings of a 31.6% increase in the total oropharyngeal volume. On the contrary, Iwasaki et al^21^ reported a 139.22% increase in oropharyngeal airway volume after Herbst appliance treatment in patients with Class II malocclusion, whereas Rizk et al^23^ found a 60.97% increase after MARA treatment. The wide variation in airway changes reported in the literature may be attributed to differences in appliance designs, follow-up durations, patient ethnicity, and baseline differences in the population. For example, Iwasaki et al^21^ did not have rapid palatal expansion in any patients and followed up for approximately 42 months, whereas Rizk et al^23^ had rapid palatal expansion for all patients and followed patients up for approximately 26 months. Moreover, a previous study reported that different ethnic groups can have varying airway dimensions.^37^ Iwasaki et al^21^ studied the Japanese population, whereas Rizk et al^23^ assessed a patient population in Michigan (with unspecified ethnicity).

After the Pendex appliance removal, we did not observe any significant changes in the upper airway dimensions compared with pretreatment (T1 to T2). In the long term, the Pendex group showed a significant increase of 31.9% in total volume, 7.5% in total length, 39.9% in RP volume, and 27.1% in RP mean CSA from T1 to T3 (44.8 ± 9.6 months). The airway changes noted between T2 and T3 can be largely attributed to natural growth, as craniofacial changes after Pendex removal at T2 primarily involved dental movements rather than skeletal modifications. The upper airway dimensions are known to increase naturally during growth in children and adolescents.^55–57^ In addition, minimal effects of maxillary molar distalization on the upper airway dimensions are supported by previous literature.^58^ Serafin et al^58^ reported negligible changes in the pharyngeal airway dimensions over 8 months in adolescent patients between pre and postpendulum molar distalization using lateral cephalometric analysis. Similarly, we followed up on the patients treated with the Pendex appliance for 13 months from T1 to T2 and found insignificant changes across all the upper airway dimensions. In addition, other methods of maxillary molar distalization, such as those assisted by temporary skeletal anchorage devices, are also associated with negligible increases in total airway volume and minimum CSA in adolescent patients, as assessed by CBCT evaluations.^29^ Chou et al^29^ reported no significant differences in the upper airway dimensions between the distalization group and the dental Class II control group. These findings align with our results, indicating that maxillary molar distalization in adolescent patients produces minimal upper airway changes, with its long-term effects primarily attributed to natural growth.

Rapid palatal expansion can be associated with nasopharyngeal airway changes.^28,59,60^ Zeng et al^61^ reported an increase in the lower nasal volume, but no change in pharyngeal airway volume after rapid palatal expansion in children. Similarly, Chang et al^62^ and El et al^59^ reported increased nasopharyngeal airway dimensions but no positive changes in the oropharyngeal airway from rapid palatal expansion.^51^ Moreover, after banded and bonded rapid palatal expansion, as well as bone-borne and tooth-borne rapid palatal expansion, the increases were observed in nasal cavity and nasopharyngeal volumes, whereas the oropharyngeal volume remained unchanged.^60,63^ Conversely, a study found that the RP airway volume significantly increased with rapid palatal expansion, with no other changes in the oropharyngeal airway dimensions.^64^ Almuzian et al^65^ reported a significant increase in the nasopharyngeal space, but a significant decrease in the upper RP space after bonded rapid palatal expansion. So far, most studies support that rapid palatal expansion may be associated with significant increases in the nasal cavity and nasopharyngeal volume, consequently decreasing nasal airway resistance. However, the effect of rapid palatal expansion on the oropharyngeal airway volume is negligible.^59,60^ Therefore, the inclusion of rapid palatal expansion in the appliance design in our study is unlikely to impact our comparison, particularly because all patients in both groups underwent rapid palatal expansion.

Head posture can influence upper airway volume.^66–68^ To obtain reproducible and reliable CBCT images for accurate airway measurements, it is recommended that images be acquired in the natural head position.^66,67,69,70^ Natural head position is defined as a reproducible posture with the head upright and eyes focused on a point at eye level. Coppelson et al^67^ reported that tilting the head downward significantly decreased the minimum CSA of the upper airway by 41.4 ± 18.5 mm^2^, whereas tilting the head upward increased it by 147.4 ± 43.3 mm^2^. These findings were further supported 3-dimensionally by Gurani et al,^71^ who observed increased upper airway volume with an upward head tilt and decreased volume with a downward head tilt.

Our study has several limitations. First, there was a significant difference in the maxillomandibular relationship between the 2 groups at T1. The Herbst group had an average ANB angle of 5.20° ± 1.89°, whereas the Pendex group had an ANB angle of 3.47° ± 1.83° (*P* = 0.002). This difference is expected, as clinicians typically use functional appliances for growth modification in patients with more pronounced skeletal Class II discrepancies rather than relying solely on intra-arch dental camouflage. To account for initial differences, statistical analyses were adjusted for the baseline T1 values. Second, all patients in both groups underwent rapid palatal expansion. However, current literature indicates that rapid palatal expansion primarily affects the nasal cavity and nasopharyngeal airway, with minimal impact on the oropharyngeal airway.^72–74^ As our study focuses on oropharyngeal airway measurements and both groups underwent rapid palatal expansion, the impact of rapid palatal expansion on the upper airway dimensions is expected to be minimal. Third, we could not directly compare immediate treatment effects because of differing durations from T1 to T2 between the groups, which were dictated by specific appliance treatment protocols beyond our control. Hence, our comparisons primarily focused on the total treatment period (T1 to T3), along with individual time-point analyses at T1 and T3. The changes observed from T1 to T3 represent a combination of natural growth and comprehensive orthodontic treatment, in addition to the effects of the Herbst and Pendex appliances. In addition, to account for variations in treatment duration, statistical analyses were adjusted for the follow-up interval between T1 and T3. Fourth, our sample size of 25 patients per group is relatively modest. Consequently, nonsignificant findings related to smaller effect sizes should be interpreted cautiously because of the potential for false negatives. Fifth, CBCT scans were not consistently performed at a specific point in the respiratory cycle, such as during inspiration or expiration, because of the limitations inherent in this retrospective study design. Sixth, our study was limited to anatomic measurements derived from CBCT images, rather than functional assessments, such as polysomnography or spirometry, potentially limiting the clinical significance of our findings. In addition, the data were obtained from a private orthodontic practice in which routine medical histories do not include body mass index information, a factor known to correlate with airway dimensions and morphology, or information regarding a history of tonsillectomy, adenoidectomy, or sleep-related breathing issues, such as snoring or apnea.^75^ Lastly, the retrospective and nonrandomized nature of our study, combined with data collection from a single private practice, introduces inherent selection bias. To minimize confounding factors and provide stronger evidence, future prospective randomized clinical trials are warranted.

## CONCLUSIONS

After Herbst appliance treatment, a significant increase in the upper airway dimensions was observed. Similarly, Pendex appliance treatment resulted in notable improvements over a comparable follow-up period, likely because of natural growth. However, when comparing the overall changes between the Herbst and Pendex groups, no statistically significant differences were found in the upper airway caliber changes between the 2 groups. Thus, the influence of Herbst appliance treatment on the upper airway dimensions appears minimal or negligible compared with the effects attributed to natural growth. These findings should be interpreted with caution, as the overall changes reflect not only the effects of the Herbst and Pendex appliances, but also the combined influence of natural growth and comprehensive orthodontic treatment.

## Figures and Tables

**Table I.** Demographics

	Herbst group (n = 25)	Pendex group (n = 25)	P value
Sex	13 M, 12 F	12 M, 13 F	0.777
Age at T1 (y)	12.16 ± 1.89	11.72 ± 1.22	0.332
ANB (°) at T1	5.20 ± 1.89	3.47 ± 1.83	0.002*
SN-GoGn (°) at T1	30.15 ± 5.92	30.82 ± 4.18	0.634^†^
Age at T3 (y)	15.59 ± 1.91	15.46 ± 1.19	0.768
ANB (°) at T3	3.22 ± 2.10	2.50 ± 2.33	0.260
SN-GoGn (°) at T3	29.52 ± 7.12	29.84 ± 4.40	0.851
Treatment period (mo)			
T1-T2	29.22 ± 9.80	14.77 ± 8.15	<0.001*
T2-T3	11.89 ± 7.86	30.03 ± 13.43	<0.001*
T1-T3	41.11 ± 9.95	44.81 ± 9.63	0.188

*M*, male; *F*, female.

* Statistically significant at the Hochberg-adjusted α-level;

† Mann-Whitney U test–based *P* value;

all other values are derived from independent-samples *t* test.

**Table II.** Definitions of the anatomic boundaries of the upper airway

	Superior boundary	Inferior boundary	Anterior boundary	Posterior boundary
Total airway	A plane parallel to FH passing through PNS and extended to the posterior wall of the pharynx	The level of the base of the epiglottis parallel to the FH plane	The posterior aspect of the soft palate and the posterior border of the tongue	The pharyngeal wall
RP airway	A plane parallel to FH passing through PNS and extended to the posterior wall of the pharynx	A plane parallel to FH passing through the level of the most inferior aspect of the soft palate	The posterior aspect of the soft palate	The pharyngeal wall
RG airway	A plane parallel to FH passing through the level of the most inferior aspect of the soft palate	The level of the base of the epiglottis parallel to the FH plane	The posterior border of the tongue	The pharyngeal wall

*PNS*, posterior nasal spine; *FH*, Frankfort horizontal plane.

**Table III.** Three-dimensional Herbst group treatment outcomes

	T1 (n = 25)		T2 (n = 25)		*T3 (n* = *25)*		P value	Significant pairwise comparisons**
	Mean	SD	Mean	SD	Mean	SD		
Total volume (mm^3^)	12,273	4625	15,668	4659	16,155	5979	<0.001*	T2-T1*, T3-T1*
Total length (mm)	51.98	5.19	58.79	5.54	60.01	6.33	<0.001*	T2-T1*, T3-T1*
Total mean CSA (mm^2^)	235.90	87.70	266.10	74.44	269.20	97.40	0.070	–
Total minimum CSA (mm^2^)	159.30	76.24	153.30	59.32	166.40	78.27	0.716	–
RP volume (mm^3^)	6987	2772	9272	3530	9677	4480	<0.001*	T2-T1*, T3-T1*
RP length (mm)	26.81	4.21	30.76	4.76	31.15	5.65	<0.001*	T2-T1*, T3-T1*
RP mean CSA (mm^2^)	258.20	91.45	299.00	93.15	306.20	120.80	0.011	T2-T1*, T3-T1*
RP minimum CSA (mm^2^)	180.30	85.40	183.60	78.12	196.40	97.76	0.530	–
RG volume (mm^3^)	5286	2407	6396	2255	6478	2716	0.023^†^	T2-T1
RG length (mm)	25.18	6.02	28.03	4.85	28.86	5.93	<0.001*	T2-T1*, T3-T1*
RG mean CSA (mm^2^)	213.20	88.64	227.30	65.54	226.20	78.40	0.604^†^	–
RG minimum CSA (mm^2^)	168.20	77.00	171.30	55.90	178.10	71.41	0.537^†^	–

*SD*, standard deviation.

* Statistically significant at the Hochberg-adjusted α-level;

** Pairwise comparisons performed if ANOVA or Friedman *P* <0.05;

† Friedman-based *P* value;

all other values are derived from repeated-measures ANOVA.

**Table IV.** Three-dimensional Pendex group treatment outcomes

	T1 (n = 25)		T2 (n = 25)		T3 (n = 25)		P value	Significant pairwise comparisons**
	Mean	SD	Mean	SD	Mean	SD		
Total volume (mm^3^)	13,763	7335	13,831	6086	18,154	7422	0.023^†^	T3-T1*, T3-T2*

Total length (mm)	54.40	5.43	55.07	5.18	58.48	5.75	0.031^†^	T3-T1*, T3-T2*

Total mean CSA (mm^2^)	249.70	116.60	251.60	110.00	307.80	110.40	0.023^†^	T3-T1*, T3-T2

Total minimum CSA (mm^2^)	166.90	105.60	148.00	89.64	211.90	100.50	0.051^†^	–

RP volume (mm^3^)	7679	3402	7908	3167	10,745	4499	0.004*^,†^	T3-T1*, T3-T2*

RP length (mm)	27.86	3.60	28.01	3.61	30.31	5.30	0.009	T3-T1*, T3-T2*

RP mean CSA (mm^2^)	279.00	120.60	284.20	111.00	354.60	119.90	0.002*	T3-T1*, T3-T2*

RP minimum CSA (mm^2^)	205.80	113.30	191.40	112.80	256.70	103.30	0.002*^,†^	T3-T2*

RG volume (mm^3^)	6083	4235	5922	3235	7408	3257	0.039^†^	T3-T1

RG length (mm)	26.53	5.40	27.06	5.24	28.16	3.49	0.304	–

RG mean CSA (mm^2^)	218.90	121.80	219.00	116.40	262.00	110.90	0.333^†^	–

RG minimum CSA (mm^2^)	173.00	106.50	160.30	99.52	220.30	108.10	0.141^†^	–

*SD*, standard deviation.

* Statistically significant at the Hochberg-adjusted α-level;

** Pairwise comparisons performed if ANOVA or Friedman *P* <0.05;

† Friedman-based *P* value;

all other values are derived from ated-measures ANOVA.

**Table V.** Three-dimensional comparison of Herbst and Pendex groups at T1

	Herbst group (n = 25)		Pendex group (n = 25)		Effect size	P value
	Mean	SD	Mean	SD		
Total volume (mm^3^)	12,273	4625	13,763	7335	−0.04	0.808*

Total length (mm)	51.98	5.19	54.40	5.43	−0.45	0.115

Total mean CSA (mm^2^)	235.90	87.70	249.70	116.60	−0.01	0.946*

Total minimum CSA (mm^2^)	159.30	76.24	166.90	105.60	−0.08	0.773

RP volume (mm^3^)	6987	2772	7679	3402	−0.09	0.580*

RP length (mm)	26.81	4.21	27.86	3.60	−0.27	0.345

RP mean CSA (mm^2^)	258.20	91.45	279.00	120.60	−0.19	0.494

RP minimum CSA (mm^2^)	180.30	85.40	205.80	113.30	−0.25	0.374

RG volume (mm^3^)	5286	2407	6083	4235	−0.05	0.749*

RG length (mm)	25.18	6.02	26.53	5.40	−0.24	0.406

RG mean CSA (mm^2^)	213.20	88.64	218.90	121.80	0.04	0.823*

RG minimum CSA (mm^2^)	168.20	77.00	173.00	106.50	−0.05	0.857

Note. No *P* values were statistically significant at the Hochberg-adjusted α-level. Effect size: Cohen *d* for t-test (small: 0.2, medium: 0.5, and large: 0.8) and Cliff delta for the Mann-Whitney U test (small: 0.15, medium: 0.33, and large: 0.47). Effect size calculated as Herbst – Pendex. *SD*, standard deviation.

* Mann-Whitney U test–based *P* value;

all other values are derived from the independent-samples *t* test.

**Table VI.** Three-dimensional comparison of Herbst and Pendex groups at T3, adjusted for baseline (T1) values of the modeled variable and follow-up duration between T1 and T3

	Herbst group (n = 25)		Pendex group (n = 25)		Effect size	P value
	Mean	95% CI	Mean	95% CI		
Total volume (mm^3^)	16451.36	13,873.91, 19,028.81	17,857.96	15,280.51, 20,435.42	−0.22	0.449

Total length (mm)	60.32	57.82, 62.82	58.16	55.66, 60.66	0.36	0.241

Total mean CSA (mm^2^)	270.82	232.18, 309.46	306.15	267.51, 344.80	−0.37	0.205

Total minimum CSA (mm^2^)	165.70	131.38, 200.03	212.56	178.24, 246.89	−0.56	0.061

RP volume (mm^3^)	9973.53	8322.87, 11,624.19	10,449.26	8798.60, 12,099.92	−0.12	0.069

RP length (mm)	31.63	29.73, 33.53	29.83	27.93, 31.73	0.39	0.189

RP mean CSA (mm^2^)	310.48	266.24, 354.72	350.34	306.10, 394.57	−0.37	0.212

RP minimum CSA (mm^2^)	199.41	161.94, 236.87	253.62	216.16, 291.08	−0.59	0.048

RG volume (mm^3^)	6508.14	5373.79, 7642.48	7378.4	6244.06, 8512.75	−0.31	0.029

RG length (mm)	29.07	27.32, 30.83	27.95	26.20, 29.70	0.26	0.376

RG mean CSA (mm^2^)	225.25	188.88, 261.62	262.92	226.55, 299.29	−0.42	0.151

RG minimum CSA (mm^2^)	176.45	141.38, 211.53	221.98	186.90, 257.05	−0.53	0.074

Note. No *P* values were statistically significant at the Hochberg-adjusted α-level. All *P* values reflect group differences from covariate-adjusted linear regression models. Effect size is calculated as standardized mean difference = adjusted group difference ÷ model residual standard deviation (root mean square error); similar to Cohen *d*, but based on regression-adjusted estimates. Interpretation: small (0.2), medium (0.5), and large (0.8). Effect size is calculated as Herbst – Pendex.

**Table VII.** Three-dimensional comparison of Herbst and Pendex groups at T3-T1, adjusted for baseline (T1) values of the modeled variable and follow-up duration between T1 and T3

	Herbst T3-T1 (n = 25)			Pendex T3-T1 (n = 25)			Effect size	P value
	Mean	95% CI	P value**	Mean	95% CI	P value**		
Total volume (mm^3^)	3433.52	856.07, 6010.97	0.010*	4840.12	2262.67, 7417.57	<0.001*	−0.22	0.449

Total length (mm)	7.13	4.63, 9.63	<0.001*	4.97	2.47, 7.47	<0.001*	0.36	0.241

Total mean CSA (mm^2^)	28.00	−10.64, 66.64	0.151	63.34	24.69, 101.98	0.002*	−0.37	0.205

Total minimum CSA (mm^2^)	2.62	−31.70, 36.95	0.879	49.48	15.16, 83.81	0.006*	−0.56	0.061

RP volume (mm^3^)	2640.39	989.73, 4291.05	0.002*	3116.12	1465.46, 4766.78	<0.001*	−0.12	0.688

RP length (mm)	4.30	2.40, 6.20	<0.001*	2.50	0.60, 4.40	0.011*	0.39	0.189

RP mean CSA (mm^2^)	41.90	−2.33, 86.14	0.063	81.76	37.52, 126.00	0.001*	−0.37	0.212

RP minimum CSA (mm^2^)	6.34	−31.12, 43.81	0.735	60.56	23.09, 98.02	0.002*	−0.59	0.048

RG volume (mm^3^)	823.44	−310.91, 1957.78	0.151	1693.70	559.36, 2828.05	0.004*	−0.31	0.289

RG length (mm)	3.22	1.47, 4.97	0.001*	2.10	0.34, 3.85	0.020*	0.26	0.376

RG mean CSA (mm^2^)	9.18	−27.19, 45.55	0.614	46.85	10.48, 83.22	0.013*	−0.42	0.151

RG minimum CSA (mm^2^)	5.87	−29.21, 40.94	0.738	51.39	16.31, 86.46	0.005*	−0.53	0.074

Note. All *P* values reflect group differences from covariate-adjusted linear regression models. Effect size is calculated as standardized mean difference = adjusted group difference ÷ model residual standard deviation (root mean square error); similar to Cohen *d*, but based on regression-adjusted estimates. Interpretation: small (0.2), medium (0.5), and large (0.8). Effect size is calculated as: (T3-T1 change in Herbst) – (T3-T1 change in Pendex).

* *P* values are statistically significant at the Hochberg-adjusted α-level;

** *P* value from within-group analyses comparing T3 vs T1.

**Table VIII.** Two-dimensional comparison of Herbst, Pendex, and Class II untreated control groups at T1

	Herbst group (n = 25)		Pendex group (n = 25)		Control group (n = 25)		Effect size	P value	Significant pairwise comparison*
	Mean	SD	Mean	SD	Mean	SD			
Total area (mm^2^)	579.67	171.33	647.47	227.49	552.89	120.27	−0.34	0.162	–

Total length (mm)	51.94	4.99	52.72	5.90	56.95	5.93	−0.14	0.005**	H-C**, P-C**

Total mean AP (mm)	11.19	3.33	12.18	3.47	9.77	2.24	−0.29	0.024	P-C**

Total minimum AP (mm)	7.10	3.51	7.37	4.10	5.86	2.27	−0.07	0.247	–

RP area (mm^2^)	335.74	119.76	355.43	94.61	283.08	78.42	−0.18	0.033	P-C

RP length (mm)	28.36	4.45	27.14	4.15	24.87	4.28	0.28	0.018	H-C**

RP mean AP (mm)	11.81	3.56	13.14	2.97	11.37	2.51	−0.41	0.106	P-C

RP minimum AP (mm)	8.47	3.45	9.70	3.48	8.58	2.10	−0.35	0.305	–

RG area (mm^2^)	243.93	87.59	292.04	154.48	269.81	78.77	−0.38	0.322	–

RG length (mm)	23.58	5.07	25.58	5.02	32.08	6.05	−0.40	<0.001**	H-C**, P-C**

RG mean AP (mm)	10.57	3.64	11.13	4.56	8.49	2.26	−0.14	0.030	H-C, H-P

RG minimum AP (mm)	7.80	3.91	8.20	4.88	5.92	2.32	−0.09	0.089	–

Note. All omnibus *P* values are from ANOVA (Welch test). Effect size represents Cohen *d* for the Herbst-Pendex comparison (small ≈ 0.2, medium ≈ 0.5, and large ≈ 0.8), calculated as (mean Herbst – mean Pendex) ÷ pooled SD.

*SD*, standard deviation.

* Pairwise comparisons performed if ANOVA omnibus *P* <0.05;

** *P* values and pairwise comparisons are statistically significant at the Hochberg-adjusted α-level.

**Table IX.** Two-dimensional comparison of Herbst, Pendex, and Class II untreated control groups at T3, adjusted for baseline (T1) values of the modeled variable and follow-up duration between T1 and T3

	Herbst group (n = 25)		Pendex group (n = 25)		Control group (n = 25)		Effect size	P value	Significant pairwise comparison*
	Mean	95% CI	Mean	95% CI	Mean	95% CI			
Total area (mm^2^)	687.28	626.80, 747.75	738.11	675.78, 800.44	659.51	598.54, 720.48	−0.34	0.214	–

Total length (mm)	59.48	56.92, 62.05	56.28	53.76, 58.80	62.78	60.17, 65.39	0.51	0.003**	P-C**

Total mean AP (mm)	11.46	10.44, 12.49	13.13	12.07, 14.20	10.87	9.81, 11.92	−0.65	0.013**	H-P, P-C**

Total minimum AP (mm)	7.16	6.12, 8.20	8.86	7.81, 9.91	6.29	5.24, 7.34	−0.66	0.004**	H-P, P-C**

RP area (mm^2^)	401.10	364.65, 437.56	407.59	370.10, 445.08	340.85	303.26, 378.45	−0.07	0.031	H-C, P-C

RP length (mm)	31.59	29.63, 33.55	28.88	26.94, 30.81	28.05	26.07, 30.04	0.57	0.039	H-C**

RP mean AP (mm)	12.76	11.30, 14.21	14.72	13.22, 16.22	12.19	10.72, 13.66	−0.54	0.056	P-C

RP minimum AP (mm)	8.80	7.71, 9.89	10.36	9.25, 11.47	8.92	7.83, 10.01	−0.58	0.100	–

RG area (mm^2^)	284.88	246.13, 323.64	332.67	293.55, 371.79	317.80	279.59, 356.01	−0.50	0.225	–

RG length (mm)	27.22	25.01, 29.43	27.19	25.12, 29.26	35.61	33.29, 37.93	0.01	<0.001**	H-C**, P-C**

RG mean AP (mm)	9.98	8.90, 11.06	11.97	10.86, 13.07	9.67	8.56, 10.78	−0.74	0.011**	H-P**, P-C**

RG minimum AP (mm)	7.59	6.52, 8.66	9.32	8.23, 10.40	6.68	5.59, 7.77	−0.65	0.005**	H-P, P-C**

Note. All *P* values reflect group differences from covariate-adjusted linear regression models (adjusted for the T1 baseline of the same variable and follow-up duration). Effect size is calculated as standardized mean difference = adjusted group difference ÷ model residual standard deviation (root mean square error); similar to Cohen *d*, but based on regression-adjusted estimates. Interpretation: small (0.2), medium (0.5), and large (0.8). Effect size is calculated as Herbst – Pendex.

* Pairwise comparisons performed if ANOVA omnibus *P* <0.05;

** *P* values and pairwise comparisons are statistically significant at the Hochberg-adjusted α-level.

**Table X.** Two-dimensional comparison of Herbst, Pendex, and Class II untreated control groups at T3-T1, adjusted for baseline (T1) values of the modeled variable and follow-up duration between T1 and T3

	Herbst T3-T 1 (n = 25)			Pendex T3-T 1 (n = 25)			Control T3-T 1 (n = 25)			Effect Size	ANCOVA P value	Significant pairwise comparison*
	Mea n 95% CI	P value**	Mean	95% CI	P value**	Mean	95% CI	P value**				
Total area (mm^2^)	93.93	33.46, 154.41	0.003***	144.77	82.44, 207.10	<0.001***	66.17	5.20, 127.13	0.034	−0.34	0.214	–
Total length (mm)	5.61	3.04, 8.18	<0.001***	2.41	−0.11, 4.93	0.060	8.91	6.30, 11.52	<0.001***	0.51	0.003***	P-C***
Total mean AP (mm)	0.42	−0.60, 1.44	0.418	2.09	1.03, 3.15	<0.001***	−0.18	−1.24, 0.88	0.735	−0.65	0.013***	H-P, P-C***
Total minimum AP (mm)	0.38	−0.66, 1.42	0.466	2.08	1.03, 3.13	<0.001***	−0.48	−1.53, 0.56	0.360	−0.66	0.004***	H-P, P-C***
RP area (mm^2^)	76.35	39.90, 112.81	<0.001***	82.84	45.35, 120.33	<0.001***	16.10	−21.49, 53.70	0.396	−0.07	0.031	H-C, P-C
RP length (mm)	4.80	2.84, 6.76	<0.001***	2.08	0.14, 4.02	0.036***	1.26	−0.72, 3.25	0.209	0.57	0.039	H-C***
RP mean AP (mm)	0.65	−0.81, 2.11	0.376	2.62	1.12, 4.12	0.001***	0.09	−1.38, 1.55	0.906	−0.54	0.056	P-C
RP minimum AP (mm)	−0.12	−1.21, 0.98	0.830	1.45	0.33,2.56	0.012***	0.00	−1.09, 1.09	0.999	−0.58	0.100	–
RG area (mm^2^)	16.29	−22.47, 55.05	0.405	64.08	24.96, 103.20	0.002***	49.21	11.00, 87.41	0.012***	−0.50	0.225	–
RG length (mm)	0.14	−2.07, 2.35	0.899	0.11	−1.96, 2.18	0.913	8.53	6.21, 10.85	<0.001***	0.01	<0.001***	H-C***, P-C***
RG mean AP (mm)	−0.08	−1.16, 1.00	0.878	1.90	0.80, 3.01	0.001***	−0.39	−1.50, 0.72	0.488	−0.74	0.011***	H-P***, P-C***
RG minimum AP (mm)	0.28	−0.79, 1.35	0.599	2.01	0.92, 3.10	<0.001***	−0.63	−1.72, 0.46	0.254	−0.65	0.005***	H-P, P-C***

Note. All *P* values reflect group differences from covariate-adjusted linear regression models. Effect size is calculated as standardized mean difference = adjusted group difference ÷ model residual standard deviation (root mean square error); similar to Cohen *d*, but based on regression-adjusted estimates. Interpretation: small (0.2), medium (0.5), and large (0.8). Effect size is calculated as (T3-T1 change in Herbst) – (T3-T1 change in Pendex).

*ANCOVA*, analysis of covariance.

* Pairwise comparisons performed if ANCOVA omnibus *P* <0.05;

** *P* value from within-group analyses comparing T3 vs T1;

*** *P* values and pairwise comparisons are statistically significant at the Hochberg-adjusted α-level.
